# Genome-wide association study of pancreatic fat: The Multiethnic Cohort Adiposity Phenotype Study

**DOI:** 10.1371/journal.pone.0249615

**Published:** 2021-07-30

**Authors:** Samantha A. Streicher, Unhee Lim, S. Lani Park, Yuqing Li, Xin Sheng, Victor Hom, Lucy Xia, Loreall Pooler, John Shepherd, Lenora W. M. Loo, Burcu F. Darst, Heather M. Highland, Linda M. Polfus, David Bogumil, Thomas Ernst, Steven Buchthal, Adrian A. Franke, Veronica Wendy Setiawan, Maarit Tiirikainen, Lynne R. Wilkens, Christopher A. Haiman, Daniel O. Stram, Iona Cheng, Loïc Le Marchand

**Affiliations:** 1 University of Hawaii Cancer Center, University of Hawaii at Mānoa, Honolulu, Hawaii, United States of America; 2 Department of Epidemiology and Biostatistics, University of California – San Francisco, San Francisco, California, United States of America; 3 Center for Genetic Epidemiology, Department of Preventive Medicine, Keck School of Medicine, University of Southern California, Los Angeles, California, United States of America; 4 Department of Epidemiology, University of North Carolina at Chapel Hill, Chapel Hill, North Carolina, United States of America; 5 University of Maryland School of Medicine, Baltimore, Maryland, United States of America; Case Western Reserve University School of Medicine, UNITED STATES

## Abstract

Several studies have found associations between higher pancreatic fat content and adverse health outcomes, such as diabetes and the metabolic syndrome, but investigations into the genetic contributions to pancreatic fat are limited. This genome-wide association study, comprised of 804 participants with MRI-assessed pancreatic fat measurements, was conducted in the ethnically diverse Multiethnic Cohort-Adiposity Phenotype Study (MEC-APS). Two genetic variants reaching genome-wide significance, rs73449607 on chromosome 13q21.2 (Beta = -0.67, P = 4.50x10^-8^) and rs7996760 on chromosome 6q14 (Beta = -0.90, P = 4.91x10^-8^) were associated with percent pancreatic fat on the log scale. Rs73449607 was most common in the African American population (13%) and rs79967607 was most common in the European American population (6%). Rs73449607 was also associated with lower risk of type 2 diabetes (OR = 0.95, 95% CI = 0.89–1.00, P = 0.047) in the Population Architecture Genomics and Epidemiology (PAGE) Study and the DIAbetes Genetics Replication and Meta-analysis (DIAGRAM), which included substantial numbers of non-European ancestry participants (53,102 cases and 193,679 controls). Rs73449607 is located in an intergenic region between *GSX1* and *PLUTO*, and rs79967607 is in intron 1 of *EPM2A*. *PLUTO*, *a lncRNA*, regulates transcription of an adjacent gene, *PDX1*, that controls beta-cell function in the mature pancreas, and *EPM2A* encodes the protein laforin, which plays a critical role in regulating glycogen production. If validated, these variants may suggest a genetic component for pancreatic fat and a common etiologic link between pancreatic fat and type 2 diabetes.

## Introduction

Pancreatic fat accumulation (also referred to as pancreatic steatosis or pancreatic lipomatosis) was first described in the 1920s. Due to difficulties in obtaining pancreatic specimens, the effect of pancreatic fat on health outcomes began only to be explored over the last decade when new imaging modalities including ultrasonography (US), computed tomography (CT), and magnetic resonance imaging (MRI) have allowed researchers to non-invasively visualize internal organs [1–4]. Although diagnostic error rates from imaging machine variability and operator errors are factors for all types of data collection, MRI has emerged as the most sensitive non-invasive method for detection and quantification of pancreatic fat [3–5].

Pancreatic fat accumulation has been examined mainly in European populations [6]. In the few studies that have included non-Europeans, the amount of pancreatic fat accumulation was seen to vary by racial/ethnic groups [7, 8]. In a small study of overweight self-reported African American and Hispanic participants, African American participants were found to have a lower MRI-assessed mean percent pancreatic fat compared to Hispanic participants (P<0.0001) [7]. Additionally, in a small study of mildly obese self-reported African American, Hispanic, and white participants, magnetic resonance spectroscopy (MRS)-assessed mean pancreatic triglyceride levels were significantly lower in Black participants compared to Hispanic and white participants (P = 0.006) [8].

Recently, Singh and colleagues (2017) conducted a meta-analysis in European American populations on the association between non-alcoholic fatty pancreas disease (NAFPD) and common metabolic diseases [6]. NAFPD (defined as >6.2% pancreatic fat in individuals consuming non-excessive amounts of alcohol) was found to be strongly associated with diabetes (risk ratio (RR) = 2.08, 95% confidence interval (95% CI): 1.44–3.00), the metabolic syndrome (RR = 2.37, 95% CI = 2.07–2.71), non-alcoholic fatty liver disease (NAFLD) (RR = 2.67, 95% CI: 2.00–3.56), and hypertension (RR = 1.67, 95% CI: 1.32–2.10) [6], after adjustment for possible confounding variables.

Increased amount of pancreatic fat has been shown to be correlated with obesity and older age [9]. There is also evidence that accumulation of pancreatic fat may be unevenly distributed, with more fatty accumulation in the anterior pancreas [9]. While the pathophysiology of pancreatic fat remains to be fully elucidated, there is evidence suggesting that accumulation of pancreatic fat can occur from either the death of pancreatic acinar cells followed by adipocyte replacement, or by adipocyte infiltration of the pancreas caused by obesity [9]. In humans, studies have revealed that increased pancreatic fat content is associated with deterioration of glycemic control, but not insulin secretion [10].

Research has shown that the process of pancreatic fat infiltration and associated adverse health outcomes may be partially reversible through diet, exercise, and/or bariatric surgery [11–13], including a study that revealed reduction in pancreatic triglyceride levels only in type 2 diabetes (T2D) patients and not in normal glucose tolerance patients after bariatric surgery [13]. This finding, along with the research showing varying amounts of pancreatic fat by race/ethnicity, further raise the possibility of a genetic component for pancreatic fat accumulation that has yet to be explored. Therefore, in this study, we conducted a GWAS of pancreatic fat evaluated by MRI in the Multiethnic Cohort-Adiposity Phenotype Study (MEC-APS) and examined two identified genome-wide significant variants for association with obesity-related biomarkers in MEC-APS, and with T2D in independent populations.

## Results

The GWAS study population consisted of 804 MEC-APS study participants, including 144 African Americans, 129 European Americans, 206 Japanese Americans, 187 Latinos, and 138 Native Hawaiians (Table 1). Median overall age at clinic visit was 69.1 years (Table 1). Study participants in the lowest quartile (0.74–1.91%) of percent pancreatic fat were more likely to be African American, have the lowest mean BMI, total fat mass, visceral fat area, subcutaneous fat area, and percent liver fat, compared to participants in the three higher quartiles. Participants who were in the highest quartile (5.11–26.6%) of percent pancreatic fat were more likely to be Japanese American, have the highest mean BMI, total fat mass, visceral fat area, subcutaneous fat area, and percent liver fat compared to participants in the three lower quartiles (Table 1).

**Table 1:** Descriptive characteristics of MEC-APS subject by quartiles of percent pancreatic fat (N = 804)^a^.

	Overall (N = 804)	Quartile 1	Quartile 2	Quartile 3	Quartile 4
		0.74–1.91 Percent Pancreas Fat (n = 201)	1.92–3.22 Percent Pancreas Fat (n = 201)	3.23–5.10 Percent Pancreas Fat (n = 201)	5.11–26.6 Percent Pancreas Fat (n = 201)
Age at clinic visit, years	69.1 (67.1, 71.1)	68.5 (67.1, 70.8)	69.0 (67.1., 71.1)	69.5 (67.0, 70.9)	69.9 (67.4, 71.4)
Sex, n (%)					
Men	421 (52%)	97 (49%)	107 (53%)	107 (53%)	111 (55%)
Women	383 (48%)	103 (51%)	94 (47%)	94 (47%)	90 (45%)
Race/ethnicity, n (%)					
African American	144 (17.9%)	54 (27%)	32 (16%)	28 (14%)	26 (13%)
European American	129 (16%)	28 (14%)	24 (12%)	40 (17%)	40 (20%)
Japanese American	206 (27%)	46 (23%)	62 (31%)	36 (18%)	60 (30%)
Latino	187 (23.3%)	46 (23%)	52 (26%)	60 (30%)	28 (14%)
Native Hawaiian	138 (17.2%)	24 (12%)	28 (14%)	40 (20%)	44 (22%)
Body mass index, kg/m^2^	27.7 (24.9, 30.8)	25.5 (23.4, 29.0)	27.9 (24.8, 30.3)	27.9 (25.6, 30.9)	29.2 (26.9, 32.2)
Total fat mass, kg	24.6 (19.5, 30.1)	22.3 (17.7, 27.7)	24.3 (19.5, 30.6)	26.2 (20.3, 31.3)	26.4 (21.9, 30.9)
Visceral fat area (L1-L5), cm^2^	24.3 (19.4, 29.9)	128.6 (88.0, 176.6)	150.0 (118.8, 200.2)	172.3 (132.7, 216.1)	199.1 (147.9, 251.5)
Subcutaneous fat area (L1-L5), cm^2^	33.0 (25.9, 40.6)	179.3 (142.5, 242.7)	211.0 (155.6, 285.9)	222.6 (168.9, 298.1)	239.3 (177.9, 300.0)
Liver fat, %	4.3 (2.9, 7.5)	3.6 (2.5, 5.6)	4.4 (2.9, 8.4)	5.0 (3.2, 8.7)	5.2 (3.2, 8.2)

^a^Count (percentage) of categorical variables and median (interquartile range) of continuous variables are presented across quartiles of percent pancreatic fat.

Overall, percent pancreatic fat had weak to moderate linear correlations with total fat mass (r = 0.22), visceral fat area (r = 0.34), subcutaneous fat area (r = 0.20), and percent liver fat (r = 0.17) (S1 Table). These correlations differed slightly by race/ethnicity, but remained weak to moderate.

**Fig 1:**
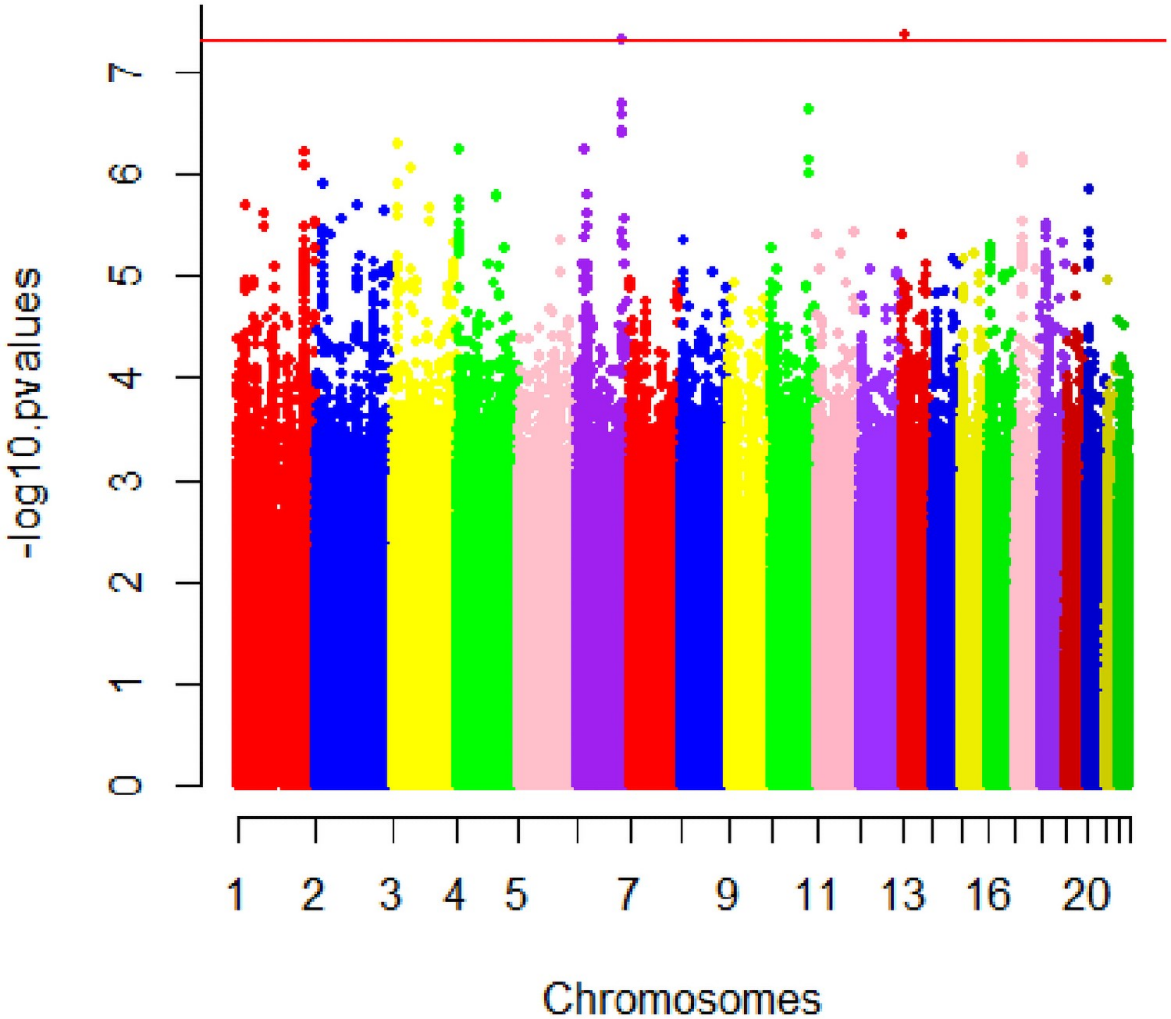
Manhattan plot of SNP P-values from the pancreas fat genome-wide association study in the Multiethnic Cohort-Adiposity Phenotype Study (MEC-APS). The Y-axis shows the negative base ten logarithm of the P-values and the X-axis shows the chromosomes. The genome-wide significance threshold, P<5x10^-8^, is shown in red.

**Fig 2:**
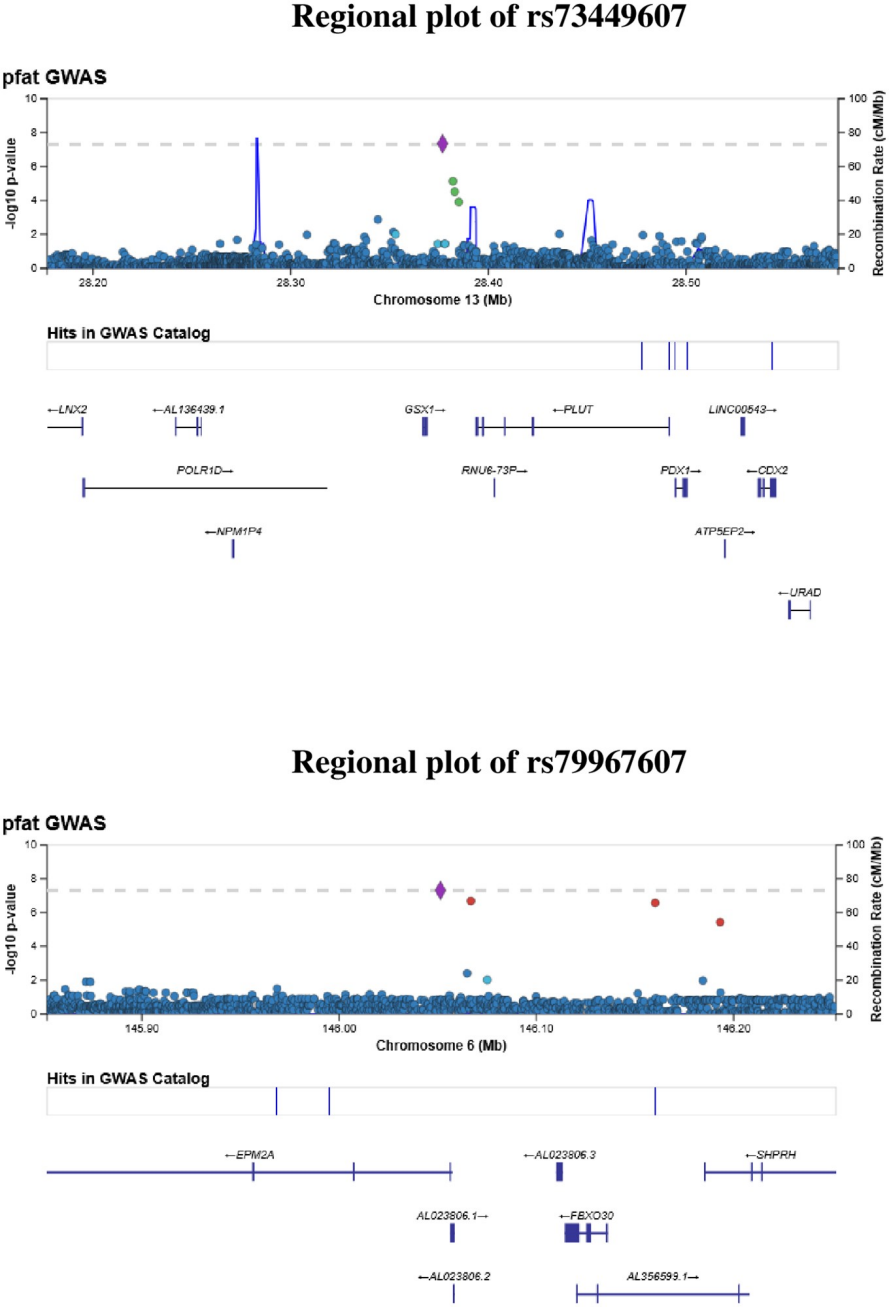
Regional plots of SNP P-values in a +/-200 kb window around rs73449607 and rs79967607. The X-axis shows the chromosome and physical location (Mb), the left Y-axis shows the negative base ten logarithm of the P-values, and the right Y-axis shows recombination activity (cM/Mb) as a blue line. Positions, recombination rates, and gene annotations are according to NCBI’s build 37 (hg 19) and the 1000 Genomes Project Phase 3 multiethnic data set.

In the MEC-APS, two loci were associated significantly with pancreatic fat at the genome-wide level: rs73449607 on chromosome 13q21.2 in an intergenic region between *GSX1* (GS Homeobox 1) and *PLUTO* (*PDX1* associated long non-coding RNA, upregulator of transcription) and rs79967607 on 6q14 in intron 1 of the *EPM2A* gene (Figs 1 and 2, Table 2). The T allele of rs73449607 on chromosome 13q21.2 was associated with a 0.49-fold (95% CI = 0.40–0.65) decrease in geometric mean percent pancreatic fat (Beta = -0.67, P = 4.50x10^-8^), independent of age, sex, and principal components (Table 2). The geometric mean percent pancreatic fat for subjects who were homozygous recessive (TT), heterozygous (TC or CT), or homozygous dominant (CC) at rs73449607 was 0.58, 1.51, or 3.05, respectively. The T allele of rs73449607 was also associated with a non-significant decrease in the odds of NAFPD (OR = 0.15; 95% CI = 0.02–1.25) (S2 Table). With additional adjustment for total fat mass, rs73449607 was associated with pancreatic fat at P = 1.62x10^-7^ (Beta = -0.27) (S3 Table). While rs73449607 had a strong association with percent pancreatic fat, weaker associations existed with total fat mass (Beta = -0.09, P = 0.05), visceral fat area (Beta = -0.13, P = 0.04), subcutaneous fat area (Beta = -0.13, P = 0.02), and percent liver fat (Beta = -0.12, P = 0.26) (S4 Table). The association between rs73449607 and percent pancreatic fat appeared to have a larger effect and smaller P-value in men compared to women, but the interaction between rs73449607 and sex was not statistically significant (P = 0.12) (Table 2). Overall, rs73449607 explained 5.3% of the variance in percent pancreatic fat. The T allele of rs73449607 was most frequent in African Americans (13%), present at low frequency in Latinos (1.1%), rare in Japanese Americans (0.2%), and not observed in Native Hawaiians or European Americans (Table 3). The most significant association across race/ethnicity between rs73449607 and percent pancreatic fat was in African Americans (Beta = -0.62; P = 9.60 × 10^−7^) with consistent effect estimates and directions of associations in the other non-monomorphic populations (Latinos and Japanese Americans) (Table 3). The interaction between the effect of rs73449607 and race/ethnicity did not reach statistical significance (P = 0.28) (Table 3). In the African American population, rs73449607 explained 14.3% of the variance in percent pancreatic fat. Overall, in PAGE/DIAGRAM, rs73449607 also showed a significant association with decreased risk of T2D (OR = 0.95; 95% CI = 0.89–1.00; P = 0.047) (Table 4). This association was driven by the African American (OR = 0.96; 95% CI = 0.90–1.02; P = 0.20) and Hispanic (OR = 0.86; 95% CI = 0.74–1.00; P = 0.047) populations (S5 Table). Of the 11 obesity-related circulating biomarkers examined in MEC-APS participants, the T allele of rs73449607 was associated with a 1.25-fold increase (Beta = 0.22; P = 1.2x10^-4^) in geometric mean for sex hormone binding globulin (SHBG) (Table 5). No association was found with other biomarkers, including glucose, insulin, or HOMA-IR (Table 5).

**Table 2:** Two genetic variants associated with percent pancreatic fat in the MEC-APS (P<5 x 10^−8^) and median (interquartile range) of percent pancreatic fat, overall and by sex.

						Overall (N = 804)				Male (n = 421)				Female (n = 383)				P-het
SNP	Chr^d^	Position^e^	Imputed Info score	Ref Allele	Effect Allele	EAF^f^	Beta^g^	SE	P-value	EAF^c^	Beta^d^	SE	P-value	EAF^c^	Beta^d^	SE	P-value	
**rs73449607**^a^^,^^b^	13	28376759	0.91	C	T	0.026	-0.67	0.12	4.50x10^-8^	0.021	-1.00	0.19	5.65x10^-7^	0.032	-0.45	0.15	0.0034	0.12
**rs79967607**^a^^,^^c^	6	146051328	0.86	T	G	0.016	-0.90	0.16	4.91x10^-8^	0.015	-0.89	0.24	2.64x10^-4^	0.018	-0.89	0.22	7.33x10^-5^	0.55
**Percent pancreatic fat**						3.2 (1.9–5.1)				3.3 (1.9–4.7)				3.1 (1.8–4.8)				

^a^Adjusted for age, sex, and principal components 1–4.

^b^For rs73449607, in the overall, male, and female population there were approximately 42, 18, and 25 T alleles, respectively.

^c^For rs79967607, in the overall, male, and female population there were 26, 13, and 14 G alleles, respectively.

^d^Chr, chromosome.

^e^Position according to NCBI build37.

^f^EAF, Effect allele frequency,

^g^Log unit change per allele increase.

**Table 3:** The association between rs73449607 or rs79967607 and pancreatic fat in the MEC-APS and median of percent pancreatic fat (interquartile range), by race/ethnicity.

		African American (n = 144)				European American (n = 129)				Japanese American (n = 206)				Latino (n = 187)				Native Hawaiian (n = 138)				P-het
SNP^a^	Chr^d^	EAF^e^	Beta^f^	SE	P	EAF^e^	Beta^f^	SE	P	EAF^e^	Beta^f^	SE	P	EAF^e^	Beta^f^	SE	P	EAF^e^	Beta^f^	SE	P	
**rs73449607**^a^^,^^b^	13	0.13	-0.62	0.13	9.60x10^-6^	-	-	-	-	0.0021	-1.38	0.9	0.13	0.011	-0.9	0.36	0.012	-	-	-	-	0.28
**rs79967607**^a^^,^^c^	6	0.018	-0.23	0.38	0.545	0.06	-1.08	0.21	1.31x10^-6^	0.0079	-1.26	0.47	7.21x10^-3^	-	-	-	-	0.0069	-1.41	0.66	0.033	0.08
**Percent pancreatic fat**		2.4 (1.4–4.4)				3.6 (2.0–5.6)				3.1 (2.0–5.4)				3.1 (2.0–4.1)				4.0 (2.3–6.2)				

^a^Adjusted for age, sex, and race/ethnic specific principal components 1–4.

^b^For rs73449607 in the African American, European American, Japanese American, Latino, and Native Hawaiian population there were approximately 37, 0, 1, 4, 0 T alleles, respectively.

^c^For rs79967607, in the African American, European American, Japanese American, Latino, and Native Hawaiian population there were approximately 5, 15, 0, 3, and 2 G alleles, respectively.

^d^Chr, chromosome.

^e^EAF, Effect allele frequency.

^f^Natural log unit change per allele increase.

**Table 4:** The association between rs73449607 or rs79967607 and type 2 diabetes in the Population Architecture Genomics and Epidemiology/DIAbetes Genetics Replication and Meta-analysis (PAGE/DIAGRAM) study.

SNP	Chr^b^	Position^c^	Imputed Info score^d^	Ref Allele	Effect Allele	Case EAF^e^^,^^f^	Control EAF^e^^,^^f^	OR (95% CI)^g^	P-value
**rs73449607**^a^	13	28376759	0.72–0.98	C	T	0.0257	0.0143	0.95 (0.89, 1.00)	0.0517
**rs79967607**^a^	6	146051328	0.81–0.97	T	G	0.0440	0.0487	0.96 (0.91, 1.01)	0.146

^a^Adjusted for age, sex, BMI and principal components.

^b^Chr, chromosome.

^c^Position according to NCBI build37.

^d^Imputation score is presented over a range from 24 different genotyping platforms.

^e^ EAF, Effect allele frequency.

^f^Effect allele frequencies were calculated based on the PAGE MEGA array data for African, Hispanic, Asian, and Native Hawaiian populations and the WHIMS data for European populations.

^g^OR, odds ratio; 95% CI, 95% Confidence Interval.

**Table 5:** The association between rs3449607 or rs79967607 and obesity-related biomarkers in the MEC-APS.

Variant	Biomarker	N	Beta^b^	SE	P-value
**rs73449607**^a^	HDL (mg/dL)	1822	0.015	0.050	0.78
	LDL (mg/dL)	1817	-0.022	0.047	0.63
	Total Cholesterol (mg/dL)	1823	-0.0014	0.032	0.96
	Glucose (mg/dL)	1821	-0.011	0.027	0.68
	HOMA-beta (%)	1810	-0.12	0.099	0.24
	HOMA-IR	1821	-0.057	0.081	0.48
	CRP (mg/L)	1823	-0.051	0.020	0.80
	Insulin (microU/mL)	1823	-0.062	0.073	0.40
	SHBG (nmol/L)	1816	0.22	0.057	1.25 x 10^−4^
	Triglycerides (mg/dL)	1823	0.053	0.053	0.57
	ALT (U/L)	1823	-0.054	0.056	0.34
**rs79967607**^a^	HDL (mg/dL)	1822	0.093	0.054	0.082
	LDL (mg/dL)	1817	0.017	0.05	0.72
	Total Cholesterol (mg/dL)	1823	0.033	0.17	0.33
	Glucose (mg/dL)	1821	0.040	0.29	0.17
	HOMA-beta (%)	1810	-0.14	0.10	0.16
	HOMA-IR	1821	0.019	0.086	0.82
	CRP (mg/L)	1823	0.078	0.22	0.71
	Insulin (microU/mL)	1823	-0.021	0.078	0.79
	SHBG (nmol/L)	1861	0.012	0.061	0.83
	Triglycerides (mg/dL)	1823	-0.031	0.57	0.59
	ALT (U/L)	1823	-0.0011	0.061	0.98

^a^Adjusted for age, sex, principal components 1–4, and total fat mass (kg).

^b g^Log unit change per allele increase.

The G allele of rs79967607 on chromosome 6q14 was associated with a 0.41-fold (95% CI = 0.29–0.56) decrease in geometric mean percent pancreatic fat (Beta = -0.90, P = 4.91x10^-8^) (Table 2). The geometric mean percent pancreatic fat for subjects who were heterozygous (GT or TG) or homozygous dominant (TT) at rs79967607 was 1.40 or 3.02, respectively. There was no participant homozygous recessive (GG) for rs79967607. The G allele of rs79967607 was also associated with a non-significant decrease in the odds of NAFPD (OR = 0.39; 95% CI = 0.09–1.69) (S2 Table). With additional adjustment for total fat mass, rs79967607 was associated with pancreatic fat at P = 2.81x10^-5^ (Beta = -0.29) (S3 Table). While rs79967607 was strongly associated with percent pancreatic fat, the variant showed weaker associations with total fat mass (Beta = -0.12, P = 0.05), visceral fat area (Beta = -0.13, P = 0.04), subcutaneous fat area (Beta = -0.19, P = 0.01), and percent liver fat (Beta = -0.064, P = 0.64) (S4 Table). Overall, rs79967607 explained 3.6% of the variance in percent pancreatic fat. The G allele of rs79967607 was most frequent in European Americans (6%) followed by African Americans (2%), rare in Latinos (0.8%) and Native Hawaiians (0.8%), and not observed in Japanese Americans. For the association between rs79967607 and percent pancreatic fat, the most significant association was in European Americans (Beta = -1.08; P = 1.31 × 10^−6^) with consistent effect estimates and direction of associations in the other non-monomorphic populations (African Americans, Latinos, and Native Hawaiians) (Table 3). The test for interaction between rs79967607 and race/ethnicity was not significant (P = 0.08) (Table 3). In the European American population, rs79967607 explained 11.9% of the variance in percent pancreatic fat. Overall, in PAGE/DIAGRAM, rs79967607 was not significantly associated with T2D (OR = 0.96; 95% CI = 0.91–1.01; P = 0.14) (Table 4 and S5 Table). Rs79967607 was not significantly associated with any of the 11 obesity-related circulating biomarkers (Table 5).

## Discussion

In our GWAS of pancreatic fat in a racially/ethnically diverse population, we observed genome-wide significant associations with percent pancreatic fat with rs73449607, a variant in an intergenic region on chromosome 13q21.2 and with rs79967607, a variant in intron 1 of *EPM2A* on chromosome 6q14. Both variants appear to be specific for pancreatic fat since they were only weakly associated with other total and ectopic adiposity phenotypes, all quantified using state-of-the-art imaging methods. In the overall analysis, rs73449607 was associated with decreased pancreatic fat content and decreased risk of T2D, and rs79967607 was associated with decreased pancreatic fat content, but not T2D. Imputation quality for rs73449607 and rs79967607 was high, and estimates and P-values obtained from regressing percent pancreatic fat on retained imputed dosages (rs73449607: Beta = -0.67, P = 4.50x10^-8^ and rs79967607: Beta = -0.90, P = 4.91x10^-8^) were almost identical to the estimates and P-values obtained from regressing percent pancreatic fat on genotypes (rs73449607: Beta = -0.63, P = 1.74x10^-8^ and rs79967607: Beta = -0.84, P = 4.52x10^-8^), adjusted for age, sex, and principal components 1–4. The T allele of rs73449607 was associated with a 49% decrease in geometric mean percent pancreatic fat, and the G allele of rs7996707 was associated with a 41% decrease in geometric mean percent pancreatic fat, after adjustment for age, sex, and principal components. Neither variant showed extreme Hardy-Weinberg departures.

In this GWAS, the first four principal components were sufficient to differentiate among the varying ethnicities in MEC. Wang and colleagues (2010) showed that African American, European American, and Japanese American MEC participants segregate on principal components 1 and 2, while Latinos separate on principal component 3, and Native Hawaiians separate on principal component 4 [14]. Moreover, we conducted the pancreatic fat GWAS adjusted for either 4 or 6 principal components (in addition to adjustment for age and sex) and found the genomic inflation λ = 1.03 and genome-wide significance (P<5x10^-8^) for both rs73449607 and rs7996760. Since genomic inflation λ was below 1.05 with 4 principal components and did not continue to decrease when adjusting for 6 principal components, adding principal components above 4 was not necessary [15].

Since rs79967607 is intronic and rs73449607 is in an intergenic region, both variants may function to affect amount of pancreatic fat through mechanisms that regulate transcriptional activity. There were no eQTLs for either variant when querying the GTEx Portal [16]. The variant rs79967607 is located in intron 1 of the *EPM2A* gene (ENCODE Accession: EH38E2512090) in a genomic region containing transcriptional regulatory elements [17]. It is located in a cis-regulatory element containing an enrichment of histone modifications in the endocrine pancreas (H3Kme3 and H3K27ac); enrichment of CCCTC-binding factor motifs (also known as CTCF) in the tissue of the body of the pancreas; and high levels of DNase in islet precursor cells [18]. Additionally, rs79967607 is associated with enrichment of H3K4me1 epigenetic motifs in pancreatic islet cells [19]. The *EPM2A* gene encodes the protein laforin, a dual specificity phosphatase, which together with the protein malin, a ubiquitin E3 ligase, plays a critical role in regulating the production of glycogen in pancreatic acinar cells [20, 21]. Loss-of-function mutations in either *EPM2A* or *EPM2B* (the gene that encodes malin) are most notably associated with Lafora disease, a very rare autosomal recessive progressive myoclonus epilepsy [20, 21]. Loss of laforin or malin function has been seen to cause an increase of glucose transporters followed by excessive glucose uptake in the brain, cardiac myocytes, kidney, fat, and the pancreas [22]. Since rs79967607 is located in an intron, the effect allele likely does not cause a loss of laforin function, but may affect expression of the gene. When blood glucose is lower, glucagon is released [23]. The main function of glucagon is to increase blood glucose through glucogenolysis and increased gluconeogenesis; however, glycogen also effects lipid metabolism, breaking down fat through lipolysis and increasing ketone production [23]. It is plausible that in people who have the G allele of rs79967607, there is also increased secretion of glucagon. This can either increase glucose to appropriate levels or lipolize pancreatic fat for energy use, consequently reducing pancreatic fat [24]. There is also some evidence that laforin may act as a tumor suppressor protein [25].

While the variant rs73449607 is closest to *GSX1* (~7.5kb downstream), it is also upstream of *PLUTO* (also known as *HI-LNC71*, *PDX1-AS1*, and *PLUT*) (~16kb upstream) and *PDX1* (pancreatic and duodenal homeobox) (~100kb upstream). There have been at least four genome-wide significant variants located in *PDX1* or *PLUTO* found to be associated with fasting blood glucose or pancreatic cancer [26–29]. *PLUTO* has been shown to affect local 3D chromatin structure and transcription of the *PDX1* gene [30]. PDX1 is a transcription factor integral to early pancreatic development and ultimately beta-cell function [31–33]. In the mature pancreas, PDX1 is mainly restricted to endocrine cells where it regulates several genes including insulin, glucose transporter-2, and glucokinase that are essential to beta-cell function [33, 34]. GSX1 is a probable transcription factor that regulates transcriptional activity of Growth Hormone-Releasing Hormone (*GHRH*) [35]. *GHRH* encodes for a neuropeptide secreted by the hypothalamus that stimulates growth hormone (*GH*) synthesis and release in the pituitary gland. In part the discovery of GHRH was made because ectopic GHRH secretion from human pancreatic islet tumors was seen to cause ectopic acromegaly by stimulating expression of GH [36]. More recently, expression of GHRH G-protein coupled receptor (GHRHR) has been identified in pancreatic islet cells [36]. In mice, GHRH has been shown to bind to GHRHR on beta-cells causing increased and preserved insulin secretion by these cells [36]. Since the T allele of rs73449607 is only common in the African American population, it is plausible that rs73449607 is located in an unidentified regulator of *PDX1* or *GSX1*, regulating expression of these cis-genes to affect pancreatic fat content.

The variant rs73449607 was also associated with increased levels of the hormonal biomarker, SHBG. Interestingly, higher SHBG levels have been associated with a lower BMI and a decreased risk of T2D, but higher SHBG levels have also been found in advanced pancreatic cancer cases [37–39]. The association between rs73449607 and SHBG seemed to be independent of obesity since the effect estimate and P-value remained similar with (Beta = 0.09; P = 3.8x10^-4^) and without (Beta = 0.11; P = 3.3x10^-5^) adjustment for total fat mass.

Most functional analyses for genetic variants have been conducted with samples from European populations. The variant rs79967607 is most common among European Americans, and examination of public resources for functional elements revealed enhanced methylation and acetylation for the endocrine pancreas of a cis-regulatory element (ENCODE Accession: EH38E2512090) in the *EPM2A* gene [18]. This suggests that the effector transcript affected by rs79967607 is *EPM2A*. The variant rs73449607 occurs only in African Americans above 1% allele frequency. We were able to place rs73449607 in an intergenic region between *GSX1* and *PLUTO*; however, public resources did not reveal additional information about potential functional mechanisms for rs73449607.

Although partial reversal of high pancreatic fat appears possible with weight loss, this study supports a genetic component to pancreatic fat deposition, which in turn, may influence other health outcomes [11–13, 40]. While historically most GWAS have been conducted in European ancestry populations, as more ethnically/racially diverse subjects become included in research studies, new multiethnic GWASes are being conducted [41]. Several recent multiethnic GWASes, including one that examined power and rate of type I error not only found it acceptable, but also beneficial to conduct a GWAS in a multiethnic population [42, 43]. Wojcik and colleagues (2019) [42] compared a standard multiethnic GWAS approach where analysis was first stratified by self-identified race/ethnicity and then combined into a meta-analysis to a joint analysis where one GWAS was conducted with all races/ethnicities [42]. They found that the joint analysis increased power compared to the meta-analysis approach, while incidence of type I error did not increase [42]. Our findings underscore the importance of conducting genetic analyses in multiethnic populations, as the significant variants varied in frequency across racial/ethnic groups, and rs73449607 was not associated with pancreatic fat in individuals of European ancestry [42]. Another strength of our analysis is that we used highly sensitive imaging methods to assess pancreatic and other ectopic fat amounts (MRI) and total fat mass (DXA), which provided the ability to test whether associations with pancreatic fat were independent of total fat mass.

In a preprint manuscript on bioRxiv, Liu and colleagues (2020) conducted GWASes of 11 MRI-assessed abdominal organ and adiposity measurements, including pancreatic volume and percent fat based on 30,000 UK Biobank participants of White British ancestry [44]. Regarding the two genome-wide significant variants in our study, rs73449607 was not observed in the European American population in MEC-APS and rs79967607 was not found to be genome-wide significantly associated with pancreatic fat in the UK Biobank population. However, 10 other significant variants were identified as genome-wide significant (P<5x10^-8^) in UK Biobank. Of these 10 significant variants [44], one variant showed an association with percent pancreatic fat in our MEC-APS study population (rs118005033: Beta = 0.10, P = 0.01), three variants were not associated with percent pancreatic fat (rs4733612: Beta = -0.07, P = 0.16; rs2270911: Beta = 0.04, P = 0.25; and rs13040225, Beta = 0.04, P = 0.27), and the remaining six variants were not in our final data set.

Although this is the first GWAS of pancreatic fat to be conducted in a multiethnic population, limitations to our study should be considered. First, due to the post-hoc measurements of pancreatic fat, only about half of the MRI scans had interpretable pancreas images. However, participant differences in interpretable and non-interpretable pancreas images were unlikely to explain our findings since sex and genetic ancestry (as principal components) were adjusted for in regression models and the genome-wide significant variants showed similar effect allele frequencies and similar or slightly stronger parameter estimates for participants with pancreatic fat data compared to all participants when other adiposity phenotypes were examined (S4 Table). Second, the total study population with MRI-assessed percent pancreatic fat was modest in size (N = 804), and the study had limited statistical power to detect weak to moderate effects. A power analysis with 804 subject shows that a GWAS would have > 80% power to detect an association size of 1.29 for a variant with a MAF > 0.40 (at P = 5x10^-8^). Third, to our knowledge, the pancreas measurements on 30,000 participants of White British Ancestry from the UK Biobank is the only other comprehensive data set of participants with image-assessed pancreatic fat or biopsy and these are not accessible in the publically available data set, which makes replicating the association between our genome-wide significant variants and pancreatic fat challenging.

In summary, two variants, rs73449607 and rs79967607, were associated with percent pancreatic fat at the genome-wide significance level in our multiethnic GWAS. The variant rs73449607 also showed an association with blood levels of SHBG and a nominal association with T2D. Studies are needed to replicate these associations in large and diverse study populations and to identify additional variants associated with pancreatic fat. These variants, if validated, may point to biologic pathways for pancreatic fat and related health outcomes, such as T2D.

## Materials and methods

### The MEC-APS

The MEC was established in 1993–1996 to examine the association of lifestyle and genetics with cancer risk [45]. This prospective study has been following over 215,000 adult men and women living in Hawaii and California, predominately Los Angeles County. Participants are mostly from five main ethnic/racial groups (African American, Japanese American, Latino, Native Hawaiian, and European American) [45]. In 2013–2016, the MEC-APS was conducted to identify predictors of body fat distribution and obesity-related cancers, as described previously [46]. Briefly, this cross-sectional study recruited 1,861 healthy, not currently smoking, male and postmenopausal female MEC participants between 60–77 years of age, with no history of chronic hepatitis, and a body mass index (BMI) between 17.1–46.2 kg/m^2^. MEC participants were selected for the study using a stratified sampling by sex, race/ethnicity, and six BMI categories. All MEC-APS participants provided written informed consent and the study was approved by the institutional review boards (IRBs) at the University of Hawaii (CHS-#17200), University of Southern California (#HS-12-00623), and University of California, San Francisco (#17–23399) in agreement with the 1975 Helsinki Declaration. Study participants underwent an abdominal MRI and body composition assessment by whole-body dual energy X-ray absorptiometry (DXA), and completed blood collection, and self-administered questionnaires including a quantitative food-frequency questionnaire [46]. Seven participants were excluded after failing genotype quality control (QC) and 1,050 were excluded for missing percent pancreatic fat measurement. Since measurements of fat deposits in the pancreas were not originally included in the MEC-APS protocol, percent pancreatic fat measurements were ascertained post-hoc. Therefore, only about half of the MRI scans yielded interpretable pancreas images, due to differences in anatomical presentation (see below). Participants with interpretable pancreatic fat MRI images were more often men (P = 0.04), Japanese Americans, Latinos, or Native Hawaiians (P<0.0001) and had greater visceral fat area (P = 0.002) and percent liver fat (P<0.0001) compared to those with non-usable MRI (S6 Table). There were no differences between the groups with and without valid pancreatic fat analysis by age (P = 0.42), total adiposity (P = 0.32), or subcutaneous fat area (P = 0.09) (S6 Table). The final study population comprised 804 MEC-APS participants.

### Adiposity measurements

The 3T MRI scans from a Siemens TIM Trio at UH and General Electric HDx at USC were used to quantify pancreatic fat, abdominal visceral and subcutaneous fat, and liver fat. Percent pancreatic fat was determined post-hoc from a series of axial triple gradient-echo Dixon-type MRI scans (10mm slices, no gap, TE = 2.4, 3.7, and 5.0 ms, TR = 160 ms, 25° flip angle) by analyzing in-phase, out-of-phase and in-phase signals in one or two circular regions of interest (ROI 15–20 cm^2^) in the pancreas, using all slices of images where a ROI could be captured while avoiding the folding of the pancreas. The Dixon protocol was applied to measure the proton density fat fraction (PDFF) of the liver and pancreas since it has shown high accuracy when compared to histologic fat fraction. It has also shown a high correlation with MR spectroscopy but has a shorter acquisition and processing time and a significantly higher sensitivity over ultrasound or computed tomography methods [47, 48]. Additional details regarding the protocol, as well as measurement of visceral fat area, subcutaneous fat area, and percent liver fat were previously published by Lim and colleagues (2019) [46]. NAFPD (188 cases and 549 controls) was defined as pancreatic fat >5% for participants with no excessive alcohol consumption (defined as >30 g/day of alcohol in men and >20 g/day of alcohol in women) in the past year [46, 49]. Total fat mass (kg) was measured by whole-body DXA scan (Hologic Discovery A densitometer, Bedford, MA) [50].

### Obesity-related biomarkers

Selected blood biomarkers were chosen for their reported associations with obesity-caused metabolic, hormonal, and inflammation dysfunctions [51]. Fasting blood samples were collected at the time of body composition measurement, processed into components, and stored at -80°C [51]. Concentrations of biomarkers (high density lipoprotein (HDL) (mg/dL) (N = 1822), low density lipoprotein (LDL) (mg/dL) (N = 1817), total cholesterol (mg/dL) (N = 1823), glucose (mg/dL) (N = 1821), homeostasis model assessment (HOMA)-beta (%) (N = 1810), HOMA-insulin resistance (IR) (%) (N = 1821), C-reactive protein (CRP) (mg/dL) (N = 1823), insulin (microU/mL) (N = 1823), SHBG (nmol/L) (N = 1816), triglycerides (mg/dL) (N = 1823), and alanine aminotransferase (ALT) (U/L) (N = 1823) were measured in blood samples from plasma or serum: detailed assay protocols and good reproducibility have been reported previously [51]. HOMA-IR and HOMA-beta were derived from fasting glucose and insulin values [51–53]. LDL cholesterol was derived from the Friedewald equation using total cholesterol and HDL cholesterol concentrations and a valid range of triglyceride concentrations [54].

### Genotyping, quality control, and imputation

Genotyping and imputation for the MEC-APS participants have been described previously [49]. Briefly, DNA extraction from buffy coat was performed using the Qiagen QIAMP DNA kit (Qiagen Inc., Valencia, CA). DNA samples were genotyped on the Illumina expanded multi-ethnic genotyping array (MEGA^EX^) platform, which to date provides the largest coverage of variants across the genome for diverse ancestral populations [55]. Variants with a call rate <95%, variants with a replicate concordance <100% based on 39 QC replicate samples, or variants with poor clustering after visual inspection were removed. Prior to imputation, monomorphic variants, variants with call rate <98%, variants with estimated minor allele frequency that deviated by ≥20% in comparison to the corresponding ancestral group in the 1000 Genomes Project Phase 3, discordance in reported vs. genotyped sex, and insertions/deletions which are not included in the Haplotype Reference Consortium (HRC), were removed. From an initial 2,036,060 genotyped variants, 1,417,570 were available for imputation. Phasing using Eagle v2.4 and genotype imputation using Minimac v4 were performed on the University of Michigan Imputation Server with the HRC vr1.1 2016 reference panel [56, 57]. After genotype imputation for MEC-APS participants, variants with an imputation quality score of < 0.4, multiallelic variants, variants with MAF<0.01, or monomorphic variants in either NAFPD cases or controls, were excluded from all subsequent analyses. In total, 9,542,479 genotyped and imputed SNPs remained after post-imputation filtering. Principal components for ancestry adjustment were calculated with 91,762 post-QC genotyped linkage disequilibrium (LD) pruned SNPs using EIGENSOFT v7 [58]. A quantile–quantile plot of GWAS P-values indicated appropriate control of type I error, with a genomic inflation (λ) value of 1.03 (S1 Fig).

### Population Architecture Genomics and Epidemiology (PAGE) study/DIAbetes Genetics Replication and Meta-analysis (DIAGRAM)

The PAGE/DIAGRAM T2D GWAS meta-analysis has been described previously [59] and was used in this study to examine the association of our pancreatic fat GWAS hits and T2D. In brief, a total of 246,781 participants from 6 case-control studies included in PAGE (ARIC, BioME, CARDIA, MEC, SOL, and WHI) and 15 case-control studies included in DIAGRAM (deCODE, DGDG, DGI, EGCUT-370, EGCUT-OMNI, EPIC-InterAct, FHS, FUSION, GoDARTS, HPFS, KORAgen, NHS, PIVUS, RS-I, ULSAM, and WTCCC) were included in a GWAS meta-analysis. There were 8,591 T2D cases and 16,887 controls of African ancestry, 3,124 T2D cases and 4,313 controls of Asian ancestry, 9,913 T2D cases and 22,958 controls from Hispanic populations, 1,642 T2D cases and 2,152 controls of Native Hawaiian ancestry, and 29,832 T2D cases and 147,369 controls of European ancestry [59]. Twenty-seven MEC-APS T2D cases and 151 controls were also included in the PAGE/DIAGRAM study.

### Statistical analysis

Descriptive characteristics were examined in the overall study population and by quartile of percent pancreatic fat (0.074–1.91%, 1.92–3.22%, 3.23–5.10%, and 5.11–26.6%). The chi-square test was used to compare categorical variables and the one-way analysis of variance (ANOVA) test was used to compare continuous variables using R v3.6.1.

Pearson’s correlations between log-transformed percent pancreatic fat and log-transformed total fat mass (N = 793), visceral fat area (N = 799), subcutaneous fat area (N = 799), and percent liver fat (N = 801) were calculated overall, and by race/ethnicity in R v3.6.1.

Variant (as imputed dosages) associations with percent pancreatic fat were estimated using linear regressions of log-transformed percent pancreatic fat, adjusted for age, sex, and main principal components 1–4 using additive genetic models, and then rerun with additional adjustment for total fat mass. SNP associations were considered statistically significant at the genome-wide significance threshold P<5x10^-8^. Imputed dosages were converted to genotypes based on a hard call threshold of 0.49999, and geometric means of percent pancreatic fat was calculated for homozygous recessive, heterozygous, and homozygous dominant genotypes. Interactions between variants significantly associated with percent pancreatic fat and sex or race were also evaluated by adding interaction terms between the variant and sex or race/ethnicity to each model. Models were further stratified by sex (male, female) and self-reported race/ethnicity (African American, European American, Japanese American, Latino, Native Hawaiian), and adjusted for age, sex, and race or sex-specific principal components. All analyses were done in PLINK v2.0.

Variants significantly associated with percent pancreatic fat were further assessed for association with total fat mass, visceral fat area, subcutaneous fat area, and percent liver fat in MEC-APS in order to examine whether they had a broader role in adiposity accumulation. Each log-transformed adiposity phenotype was regressed on the significant variant, adjusting for age, sex, and principal components 1–4 overall (N = 1,825 for total fat mass, 1,787 for visceral fat area and subcutaneous fat area, and 1,775 for percent liver fat) and limited to participants with pancreatic fat data (N = 793 for total fat mass, 799 for visceral fat area and subcutaneous fat area, and 1,775 for percent liver fat) using R v.3.6.1.

Variation in percent pancreatic fat (R^2^) explained by each genome-wide significant variant was calculated by $\frac{{Cov(X,Y)}^{2}}{Var\left(X\right)*Var(Y)}=\frac{b^{2}Var(X)}{b^{2}Var\left(X\right)+\sigma^{2}}$, where *X* = the imputed dosage variable, *σ*^2^ = the variance of the residuals, and for a variant with the effect allele frequency *p*, *Var*(*X*) = 2*p*(1 − *p*), under the Hardy-Weinberg equilibrium (HWE) assumption.

Variants significantly associated with percent pancreatic fat were also assessed for relationships with NAFPD in MEC-APS (188 cases and 549 controls), with obesity-related biomarkers (HDL, LDL, total cholesterol, glucose, insulin, HOMA-beta, HOMA-IR, CRP, SHBG, triglycerides, and ALT) among over 1,800 MEC-APS participants (see above in *Obesity-related biomarkers* for exact number of participants analyzed for each biomarker), and with T2D among 53,102 cases and 193,679 controls in PAGE/DIAGRAM. Associations with NAFPD was assessed using logistic regression models adjusted for age, sex, total fat mass, and principal components 1–4. Associations with obesity-related biomarkers were assessed using linear regression models of log-transformed analytes adjusted for age, sex, total fat mass, and principal components 1–4. Both NAFPD and obesity-related biomarkers regression models were run in PLINK v2.0. Associations with T2D were assessed with unconditional logistic regression models adjusted for age, sex, body mass index, and principal components. Every racial/ethnic population within each T2D study was analyzed separately. Racial/ethnic population-specific estimates were obtained by combining per-allele odds ratios and standard errors across studies for each racial/ethnic population. Overall estimates were obtained by combining per-allele odds ratios and standard errors first across racial/ethnic populations within each study and then by combining per-allele odds ratios and standard errors across each study. Both racial/ethnic population-specific estimates and overall estimates were obtained using fixed-effects inverse-variance weighted meta-analyses, as implemented in METAL [59, 60].

## Supporting information

S1 Fig Q-Q plot of SNP P-values from the percent pancreas fat GWAS. The Y-axis shows the negative base ten logarithm of the observed p-values and the X-axis shows the negative base ten logarithm of the expected p-values. Genomic inflation λ = 1.03.
(DOCX)

S1 Table Pearson’s correlation coefficients between percent pancreas fat and total body fat, visceral fat area, subcutaneous fat area and percent liver fat, overall and by race/ethnicity. (XLSX)

S2 Table The association between rs73449607 or rs79967607 and non-alcoholic fatty pancreas disease (NAFPD) in 188 subjects with NAFPD and 549 controls the MEC-APS. (XLSX)

S3 Table The association between rs73449607 or rs79967607 and percent pancreas fat further adjusted for total fat mass in the MEC-APS (N = 793). (XLSX)

S4 Table The association between rs73449607 or rs79967607 and four adiposity phenotypes in all MEC-APS participants and MEC-APS participants limited to those with pancreatic fat data. (XLSX)

S5 Table The association between rs73449607 or rs79967607 and type 2 diabetes in the Population Architecture Genomics and Epidemiology/DIAbetes Genetics Replication and Meta-analysis (PAGE/DIAGRAM), by race/ethnicity. (XLSX)

S6 Table Descriptive characteristics of the Multiethnic Cohort Adiposity Phenotype Study (MEC-APS) in subjects with or without a valid pancreas scan. (XLSX)
