# Supplementary material for: Genome-wide association study of pancreatic fat: The Multiethnic Cohort Adiposity Phenotype Study
Source: PLoS One. 2021 Jul 30;16(7):e0249615. doi: 10.1371/journal.pone.0249615 (PMC8323875; doi:10.1371/journal.pone.0249615)

**S1 Fig. Q-Q plot of SNP P-values from the percent pancreas fat GWAS. The Y-axis shows the negative base ten logarithm of the observed p-values and the X-axis shows the negative base ten logarithm of the expected p-values. Genomic inflation λ=1.03**


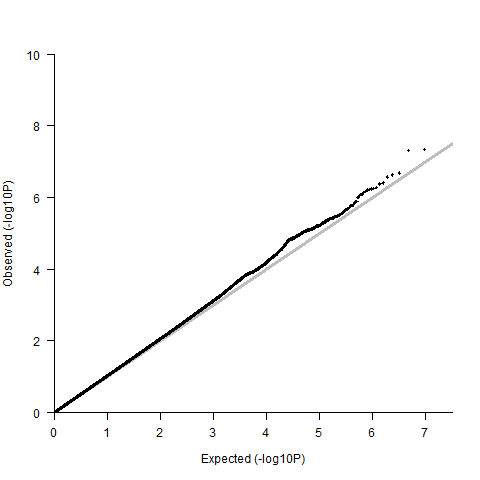

Supplement: S1 Fig — The Y-axis shows the negative base ten logarithm of the observed p-values and the X-axis shows the negative base ten logarithm of the expected p-values. Genomic inflation λ = 1.03. (DOCX) [file pone.0249615.s001.docx]
